# Molecular epidemiology and genotype/subtype distribution of *Blastocystis* sp., *Enterocytozoon bieneusi*, and *Encephalitozoon* spp. in livestock: concern for emerging zoonotic infections

**DOI:** 10.1038/s41598-021-96960-x

**Published:** 2021-09-01

**Authors:** Hanieh Mohammad Rahimi, Hamed Mirjalali, Mohammad Reza Zali

**Affiliations:** 1 Foodborne and Waterborne Diseases Research Center, Research Institute for Gastroenterology and Liver Diseases, Shahid Beheshti University of Medical Sciences, Tehran, Iran; 2 Gastroenterology and Liver Diseases Research Center, Research Institute for Gastroenterology and Liver Diseases, Shahid Beheshti University of Medical Sciences, Tehran, Iran

**Keywords:** Infectious-disease diagnostics, Parasitology

## Abstract

Intestinal parasitic infections have high prevalence rate in many regions especially in developing countries. The aim of this study was to determine the presence and genotype/subtype of some intestinal protozoa in livestock in Iran. Stool samples were collected from cattle, sheep, chickens, and horses. The presence of targeted parasites was evaluated using real-time PCR. Genotyping/subtyping of positive samples was characterized using sequencing of the ITS and barcoding region, respectively. *Blastocystis* sp., 27.7% (48/173) and *Enterocytozoon bieneusi* 26.0% (45/173) were the most frequent protozoa followed by *Encephalitozoon* spp., 0.57% (1/173). *Cryptosporidium* spp. were not detected among samples. *Encephalitozoon* spp., was detected only in chickens 2.2% (1/45). A statistically correlation was seen between animals and the prevalence of targeted protozoa. *E. bieneusi* genotypes I (9/38; 23.68%), BEB6 (22/38; 57.89%), D (6/38; 15.79%), and horse1 (1/38; 2.63%) were detected among samples. A statistically significant correlation was seen between the genotypes and animals (*P* ≤ 0.05). *Blastocystis* sp., ST1 (1/45; 2.22%), ST5 3/45; 6.66%), ST7 (1/45; 2.22%), ST10 (24/45; 53.33%), and ST14 (16/45; 35.55%) were characterized among samples. There was no significant correlation between certain subtypes and animals (*P* = 0.173). The presence of zoonotic potential genotypes of *E. bieneusi* in animals and zoonotic potential subtypes ST1 and ST7 among our samples provide a clue about the transmission dynamic of *E. bieneusi* and *Blastocystis* sp. between animals–animals and humans–animals.

## Introduction

Infections caused by intestinal parasites are still one of the important public health problems in the world. A wide range of helminths and protozoa can infect or colonize the gastrointestinal tract of humans and animals. The enteric protozoa such as *Cryptosporidium* spp., *Enterocytozoon bieneusi*, and *Encephalitozoon* spp., are of the most important zoonotic parasites causing diarrhea in humans^1^, which infect a wide range of domesticated and wild animals, as well^2^. In addition, *Blastocystis* sp. is the a prevalent protist, which its pathogenic role is still under question^3^.

These microorganisms are typically transmitted through several routes, such as direct contact with infected persons (anthroponotic transmission) or animals (zoonotic transmission), and ingestion of infective cyst/ oocyst/spore through contaminated water or food^4,5^. Asymptomatic infections due to aforementioned parasites are mostly reported from immunocompetent subjects; however, a broad range of clinical manifestations like chronic diarrhea, nausea, weight loss, vomiting, dysentery, and fever have been recorded from children, travelers, and the elderly individuals. In general, the clinical symptoms in immunocompromised individuals, especially in HIV^+^/AIDS patients are more severe^6,7^.

As for *Cryptosporidium*, 1.3 million deaths worldwide with increasing reports of diarrheal diseases has placed this protozoan as the fourth leading cause of death in children under the age of 5-years^8^. *Blastocystis* sp., is an intestinal protist, living in the digestive system of humans and a large variety of non-human hosts including non-human primates, birds, and other mammals. Several studies have shown that people with close contact to animals are more at higher risk for *Blastocystis* sp. infection^9–11^.

Microsporidia are a large and diverse group of obligatory intracellular pathogens, which can infect a broad spectrum of vertebrates and invertebrates including mammalian, birds, fishes, and insects, over the world^12–15^. Regarding the phylogenetic analysis of conserved genes, microsporidia are now reclassified as fungi^16,17^. *E. bieneusi* and *Encephalitozoon* species including (*E. cuniculi*, *E. intestinalis* and *E. hellem*) are the major species of microsporidia, infecting a wide range of mammalian hosts including humans and animals, and are responsible for almost all of the intestinal infections^18,19^. However, clinical symptoms of microsporidiosis range from self-limited diarrhea in immunocompetent subjects to disseminated infection in immunocompromised patients.

Therefore, concerning the importance of zoonotic transmission of *Cryptosporidium*, *Blastocystis* sp., *E. bieneusi*, and *Encephalitozoon* spp., the current study aimed to investigate the prevalence of these protozoa using a sensitive rapid molecular method. In addition, the genotypes and subtypes of positive cases were characterized to provide data on their host-adaptation and potentially zoonotic transmission.

## Results

Real-time PCR showed that from 173 stool samples, targeted protozoa were identified in 80 (46.2%) samples. Accordingly, *Blastocystis* sp., *E. bieneusi*, and *Encephalitozoon* spp. were detected from 27.7% (48/173), 26.0% (45/173), 0.57% (1/173) of samples, respectively (Supplementary Fig. 1). *Blastocystis* sp. and *E. bieneusi* were the most common species detected in samples. *Cryptosporidium* spp. were not detected in samples. According to results, the prevalence of the parasites in cattle was 23/32 (71.87%), followed by sheep 40/70 (57.14%), chickens 14/45 (31.11%), and horses 3/26 (11.53%). A statistically correlation was seen between types of animal and the prevalence of targeted protozoa (*P* < 0.05).

The mean ± SD of Ct values of positive samples of *Blastocystis* sp. in cattle, sheep, and chickens were shown as fallow: 28.08 ± 2.74, 27.55 ± 2.12 and 28.39 ± 2.07, respectively (Supplementary Fig. 2a). Also, real-time PCR indicated mean ± SD of T_m_ values of positive samples of *Blastocystis* sp. in these animals as fallow: 79.48 ± 0.41, 79.11 ± 0.19, and 79.60 ± 0.84 (Supplementary Fig. 3a). The prevalence rate of positive samples of *Blastocystis* sp. in cattle, sheep, and chickens was shown as fallow: 50% (16/32), 42.9% (30/70), 4.4% (2/45), respectively (Table 1). The results of statistical analysis showed that there was a statistical significant association between the presence of *Blastocystis* and the types of animal (*P* < 0.05).

**Table 1:** Intestinal parasites identified in domestic animals by real-time PCR.

Animals (no. samples)	Parasites			
	Total infection (%)	*Blastocystis* sp. (%)	*Enterocytozoon bieneusi* (%)	*Encephalitozoon* spp. (%)
Cattle (n = 32)	23 (71.87)	16 (50)	11 (34.4)	–
Sheep (n = 70)	40 (57.14)	30 (42.9)	18 (25.7)	–
Chicken (n = 45)	14 (31.11)	2 (4.4)	13 (28.9)	1 (2.2)
Horse (n = 26)	3 (11.53)	–	3 (11.5)	–
Total = 173	80 (46.2)	48	45	1

Concerning the results, mean ± SD of Ct values for positive samples of *E. bieneusi* in cattle, sheep, chickens, and horses were shown as fallow: 28.64 ± 4.26, 30.2 ± 1.84, 31.37 ± 1.15, and 29.02 ± 0.01, respectively (Supplementary Fig. 2b). Also, real-time PCR indicated mean ± SD of T_m_ values for positive samples of *E. bieneusi* in these animals as fallow: 82.30 ± 0.77, 81.71 ± 0.74, 82.63 ± 0.19, and 82.40 ± 0.17, respectively (Supplementary Fig. 3b). Furthermore, the prevalence rate of *E. bieneusi* in cattle, sheep, chickens, and horses were shown as fallow: 34.4% (11/32), 25.7% (18/70), 28.9% (13/45), 11.5% (3/26), respectively (Table 1). No statistical significant association was evidenced between the presence of *E. bieneusi* and the types of animal (*P* = 0.230).

Also, *Encephalitozoon* sp. was observed only in one chicken sample with Ct and T_m_ values of 31.72 and 84.7, respectively (Supplementary Figs. 2, 3c). Furthermore, the prevalence rate of *Encephalitozoon* sp. in chicken was 2.2% (1/45) (Table 1). No statistical significant association was evidenced between the presence of *Encephalitozoon* sp. and the types of animal (*P* = 0.595). In addition, no cases of *Cryptosporidium* spp. were detected by real-time PCR.

### *E. bieneusi* genotyping and phylogenetic analysis

**Figure 1:**
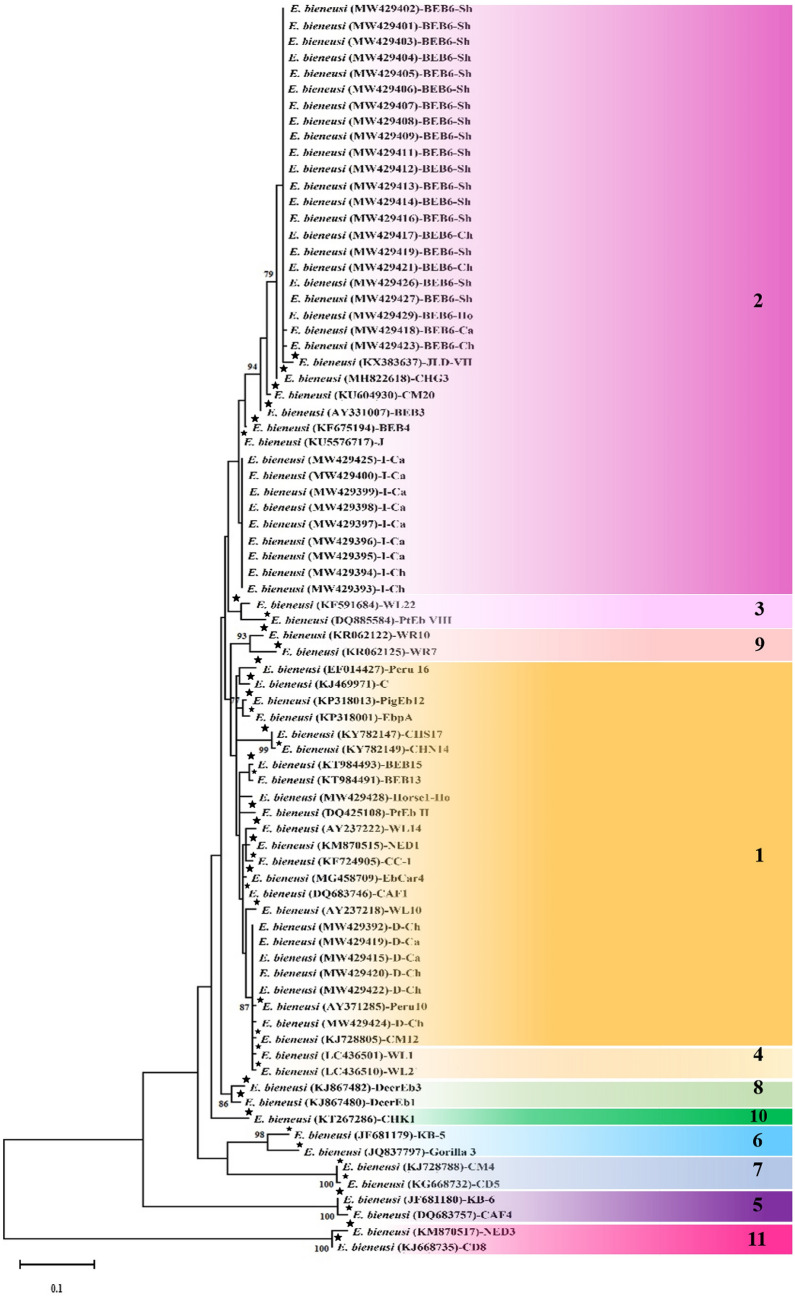
Phylogenetic tree of the ITS gene for *E. bieneusi* isolated from cattle, sheep, chicken, and horses together with reference sequences represents that all identified genotypes were cluster in two groups: 1 (1a and 1e) and 2 (2b and 2c). The phylogenetic tree was drawn using the maximum-likelihood method and the Tamura 3-parameter model. Bootstrap support (%) values of > 75% are indicated above the branches. Asterisks indicate reference genotypes. Sh: Sheep; Ca: Cattle; Chi: Chicken; Ho: Horse.

The ITS fragment of the ribosomal RNA (rRNA) gene was successfully amplified among 38/45 (84.44%) of real-time PCR-positive samples. All amplified samples were effectively sequenced and the BLAST analysis showed the presence of the genotypes I (9/38; 23.68%), BEB6 (22/38; 57.89%), D (6/38; 15.79%), and horse1 (1/38; 2.63%) among samples. A statistically significant correlation was seen between the genotypes and animals (*P* ≤ 0.05). The genotype I was reported from cattle (7/9; 77.77%) and chickens (2/9; 22.23%). The genotype BEB6 was the most prevalent genotype and was characterized from sheep (17/22; 77.27%), chickens (3/22; 13.63%), cattle (1/22; 4.54%), and horse (1/22; 4.54%). The genotype D was identified from chickens (4/6; 66.67%) and cattle (2/6; 33.33%). The genotype horse1 was only characterized from horse samples (Tables 2, 3). The phylogenetic tree of the internal transcribed spacer (ITS) fragment of *E. bieneusi* revealed that all the genotypes were clearly separated in accordance with the currently known genotypes and groups. All the genotypes D, horse1, I, and BEB6 were also clearly divided into four clusters including 1a, 1e, 2b, and 2c, respectively (Fig. 1).

**Table 2:** The genotype distribution of *E. bieneusi* among animals.

Animals	Genotype				Total (%)
	I (%)	BEB6 (%)	D (%)	Horse 1 (%)	
Cattle	7 (77.8)	1 (4.5)	2 (33.3)	–	10 (26.3)
Sheep	–	17 (77.3)	–	–	17 (44.7)
Chicken	2 (22.2)	3 (13.7)	4 (66.7)	–	9 (23.7)
Horse	–	1 (4.5)	–	1 (100)	2 (5.3)
Total	9 (23.7)	22 (57.9)	6 (15.8)	1 (2.6)	38 (100)

**Table 3:** Genotype distribution, classification, and accession numbers of *E. bieneusi-*positive samples.

Sample	Hosts	Genotypes	Groups	Accession number
EA1	Chicken	D	1a	MW429392
EA2	Chicken	I	2b	MW429393
EA3	Chicken	I	2b	MW429394
EA4	Cattle	I	2b	MW429395
EA5	Cattle	I	2b	MW429396
EA6	Cattle	I	2b	MW429397
EA7	Cattle	I	2b	MW429398
EA8	Cattle	I	2b	MW429399
EA9	Cattle	I	2b	MW429400
EA10	Sheep	BEB6	2c	MW429401
EA11	Sheep	BEB6	2c	MW429402
EA12	Sheep	BEB6	2c	MW429403
EA13	Sheep	BEB6	2c	MW429404
EA14	Sheep	BEB6	2c	MW429405
EA15	Sheep	BEB6	2c	MW429406
EA16	Sheep	BEB6	2c	MW429407
EA17	Sheep	BEB6	2c	MW429408
EA18	Sheep	BEB6	2c	MW429409
EA19	Cattle	D	1a	MW429410
EA20	Sheep	BEB6	2c	MW429411
EA21	Sheep	BEB6	2c	MW429412
EA22	Sheep	BEB6	2c	MW429413
EA23	Sheep	BEB6	2c	MW429414
EA24	Cattle	D	1a	MW429415
EA25	Sheep	BEB6	2c	MW429416
EA26	Chicken	BEB6	2c	MW429417
EA27	Cattle	BEB6	2c	MW429418
EA28	Sheep	BEB6	2c	MW429419
EA29	Chicken	D	1a	MW429420
EA30	Chicken	BEB6	2c	MW429421
EA31	Chicken	D	1a	MW429422
EA32	Chicken	BEB6	2c	MW429423
EA33	Chicken	D	1a	MW429424
EA34	Cattle	I	2b	MW429425
EA35	Sheep	BEB6	2c	MW429426
EA36	Sheep	BEB6	2c	MW429427
EA37	Horse	Horse 1	1e	MW429428
EA38	Horse	BEB6	2c	MW429429
EA39	Cattle	Not amplified	–	–
EA40	Sheep	Not amplified	–	–
EA41	Chicken	Not amplified	–	–
EA42	Chicken	Not amplified	–	–
EA43	Chicken	Not amplified	–	–
EA44	Chicken	Not amplified	–	–
EA45	Horse	Not amplified	–	–

### *Blastocystis* subtyping and phylogenetic analysis

**Figure 2:**
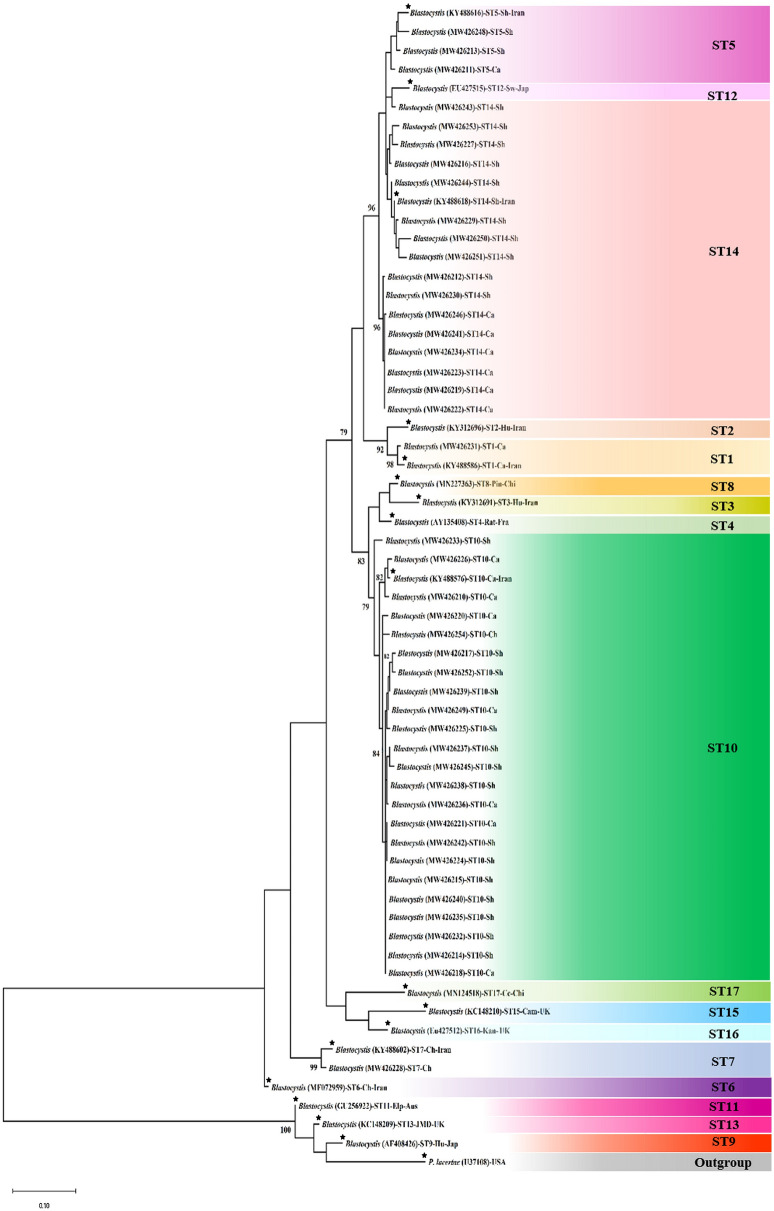
Phylogenetic tree of the barcoding fragment of *Blastocystis* sp. isolated from cattle, sheep, chicken, and horses together with reference sequences. The phylogenetic tree represents that all identified subtypes were clearly clustered. The phylogenetic tree was drawn using the maximum-likelihood method and the Tamura 3-parameter model. Bootstrap support (%) values of > 75% are indicated above the branches. Asterisks indicate reference subtypes. Jap: Japan; Chi: China; Fra: France; UK: United Kingdom; Aus: Australia; USA: United State of America; Sh: Sheep; Ca: Cattle; Sw: Swamp wallaby; Hu: Human; Chi: Chicken; Pin: Pigeon; Kan: Kangaroo; Cam: Camel; Cc: Chinchilla; Elp: Elephant; JMD: Java Mouse Deer. One of *Blastocystis* sp., ST10 with accession number MW426247 was short to be included in phylogenetic tree.

The barcoding region was successfully amplified among 47/48 (97.91%) of samples, which were *Blastocystis* sp.-positive using real-time PCR. All positive samples were sequenced that apart from two samples, all other 45 PCR products provided suitable sequencing results. Accordingly, sequencing results represented the presence of ST1 (1/45; 2.22%), ST5 3/45; 6.66%), ST7 (1/45; 2.22%), ST10 (24/45; 53.33%), and ST14 (16/45; 35.55%) among samples (Table 4). The statistical correlation between certain subtypes and animals was assessed that the results showed no significant correlation (*P* = 0.173). ST1 was only characterized from cattle. ST5 was detected from cattle (1/4; 33.33%) and sheep (2/3; 66.66%). ST7 was only reported from chicken. ST10 was the most prevalent subtype and was detected from sheep (16/24; 66.66%), cattle (7/24; 29.16%), and a chicken (1/24; 4.16%). ST14 was identified in sheep (10/16; 62.5%) and cattle (6/16; 37.5%). The allele analysis represented that ST1 was allele 4. All ST5 sequences were allele 115. ST7 showed allele 99, and all ST10 sequences exhibited allele 152 (Table 5). The phylogenetic analysis showed that all subtypes were clearly separated based on the currently known subtypes. The phylogenetic tree also revealed that there was no separation based on the hosts. In addition, similar subtypes, which were isolated from different hosts, were clustered together with bootstrap support ranging from 80 to 99% (Fig. 2).

**Table 4:** The prevalence of *Blastocystis* sp., subtype in studied animals.

Animals	Subtypes					Total (%)
	ST1 (%)	ST5 (%)	ST7 (%)	ST10 (%)	ST14 (%)	
Cattle	1 (100)	1 (33.3)	–	7 (29.2)	6 (37.5)	15 (33.3)
Sheep	–	2 (66.7)	–	16 (66.7)	10 (62.5)	28 (62.2)
Chickens	–	–	1 (100)	1 (4.1)	–	2 (4.5)
Total	1 (2.2)	3 (6.7)	1 (2.2)	24 (53.3)	16 (35.6)	45 (100)

**Table 5:** Subtype and allele distribution and accession numbers of *Blastocystis* sp.,-positive.

Sample	Host	Subtypes	Alleles	Accession number
BA1	Cattle	Low quality	–	–
BA2	Cattle	ST10	152	MW426210
BA3	Cattle	ST5	115	MW426211
BA4	Sheep	ST14	Unknown allele	MW426212
BA5	Sheep	ST5	115	MW426213
BA6	Sheep	ST10	152	MW426214
BA7	Sheep	ST10	152	MW426215
BA8	Sheep	Low quality	–	–
BA9	Sheep	ST14	Unknown allele	MW426216
BA10	Sheep	ST10	152	MW426217
BA11	Cattle	ST10	152	MW426218
BA12	Cattle	ST14	Unknown allele	MW426219
BA13	Cattle	ST10	152	MW426220
BA14	Cattle	ST10	152	MW426221
BA15	Cattle	ST14	Unknown allele	MW426222
BA16	Cattle	ST14	Unknown allele	MW426223
BA17	Sheep	ST10	152	MW426224
BA18	Sheep	ST10	152	MW426225
BA19	Cattle	ST10	152	MW426226
BA20	Sheep	ST14	Unknown allele	MW426227
BA21	Chicken	ST7	99	MW426228
BA22	Sheep	ST14	Unknown allele	MW426229
BA23	Sheep	ST14	Unknown allele	MW426230
BA24	Cattle	ST1	4	MW426231
BA25	Sheep	ST10	152	MW426232
BA26	Sheep	ST10	152	MW426233
BA27	Cattle	ST14	Unknown allele	MW426234
BA28	Sheep	ST10	152	MW426235
BA29	Cattle	ST10	152	MW426236
BA30	Sheep	ST10	152	MW426237
BA31	Sheep	ST10	152	MW426238
BA32	Sheep	ST10	152	MW426239
BA33	Sheep	ST10	152	MW426240
BA34	Cattle	ST14	Unknown allele	MW426241
BA35	Sheep	ST10	152	MW426242
BA36	Sheep	ST14	Unknown allele	MW426243
BA37	Sheep	ST14	Unknown allele	MW426244
BA38	Sheep	ST10	152	MW426245
BA39	Cattle	ST14	Unknown allele	MW426246
BA40	Sheep	ST10	152	MW426247
BA41	Sheep	ST5	115	MW426248
BA42	Sheep	Low quality	–	–
BA43	Cattle	ST10	152	MW426249
BA44	Sheep	ST14	Unknown allele	MW426250
BA45	Sheep	ST14	Unknown allele	MW426251
BA46	Sheep	ST10	152	MW426252
BA47	Sheep	ST14	Unknown allele	MW426253
BA48	Chicken	ST10	152	MW426254

## Discussion

*Blastocystis* sp., microsporidia, and *Cryptosporidium* spp., are among protozoa, which may be zoonotically transmitted to humans. In the current study 46.2% of samples were detected positive for selected parasites using real-time PCR. This prevalence rate is similar to previous reports from Belgium^20^, Canada^21^, France^22^, China^12^, but is higher than another report from China (25.6%)^23^. Although evaluated pathogens between our study and most of indicated reports are similar, it seems that method of evaluation has critical role in true estimation of the prevalence. Actually, in the study performed by Yu et. al. (2018)^23^, parasitological techniques were used to detect parasites while molecular genotyping was performed for only those samples, which were positive for *Giardia* and *Cryptosporidium*; therefore, a lower prevalence rate was expectable. In this regard, Incani et al., (2017)^24^ investigated the prevalence of intestinal parasites in a rural community and compared the results with microscopy, and concluded that real-time PCR was a more sensitive technique, although microscopy could be advisable, particularly in cases without molecular tests.

*E. bieneusi* is a prevalent microorganism in humans and animals. Increasing reports suggest the importance of zoonotic transmission of *E. bieneusi* due to the low host-specificity of most of its genotypes^18^. In the current study, *E. bieneusi* was detected from 34.4% (11/32), 25.7% (18/70), 28.9% (13/45), and 11.5% (3/26) of cattle, sheep, chickens, and horses, respectively, with an overall prevalence rate 26.0% (45/173). The prevalence of *E. bieneusi* in cattle is higher than previous reports from China^15,25,26^, Thailand^27^, Turkey^28^, Brazil^29^, Australia^30^, and the United States (USA)^31^, but it is in the line of reports from the USA^32^ and China^33,34^. The genotypes D, hoerse1, I, and BEB6 were characterized in the current study that are categorized as groups 1a, 1e, 2b, and 2c, respectively. These genotypes were all or individually reported in studies from the USA^31,32,35^, Argentina^36^, Germany^37^, Australia^30^, Thailand^27^, and many studies from China^25,34,38–40^. The genotype D is the most frequently reported genotype from humans and broad range of domesticated and wild animals^7,41–43^ and thought to be a high zoonotic potential genotype with worldwide distribution. The genotype I is one of the most prevalent genotypes in cattle, which together with the genotype BEB6, were reported from humans^44,45^, as well. These genotypes are categorized among the genotypes with low level of host specificity and increasing zoonotic concerns^18,46^. In contrast to the genotype I, which is frequently reported from cattle and thought to be a cattle genotype, the genotype BEB6 is common in sheep and was suggested that this genotype has been probably adapted to cattle during years^46,47^.

The prevalence of *E. bieneusi* in sheep is in line of some studies from China^48,49^, higher than studies from Brazil^29^, Ethiopia^50^, and lower than reports from Sweden^47^ and China^39,51^. In the line of our study, the genotype BEB6 is the most prevalent genotype reported from sheep^47,50,51^. However, this genotype represents low level of host specificity and is reported from broad range of animals, and humans^45^. The genotype BEB6 was recently reported from raw milk of sheep and cattle^52^, which increases the zoonotic concern due to the emerging of *E. bieneusi* genotype BEB6 in humans.

Reports of the prevalence of *E. bieneusi* in chickens are limited. For the first time, Reetz et al., (2002)^53^ detected *E. bieneusi* from two of six chickens in Germany. There are reports of *E. bieneusi* in chicken in the world^54–56^. Recently, *E. bieneusi* was identified in 7.3% of chicken samples in Turkey, neighboring to Iran^57^. As a result, *E. bieneusi* was detected from 28.9% of chicken samples, which is higher than previous reports in the world. The reason for this observation could be related to the method of detection. Actually, in the current study, real-time PCR was employed to detect *E. bieneusi*, which has higher sensitivity compared to conventional PCR. The genotype D, BEB6, and I were characterized in chicken samples. As mentioned above, these genotypes show low level of host specificity and have been reported from broad range of animals^18^. The genotype D was reported from chickens in a study conducted by Cao et al. (2020)^56^, and is known as the most prevalent genotypes in the world. Many studies in Iran reported this genotype from humans^7,43^, wastewater^58^, vegetables^58^, and wild and domesticated animals^13,42,59,60^, which implies the cross-transmissibility of this genotype between humans and animals and the importance of zoonotic transmission of the genotype D in Iran. The presence of the genotypes BEB6 and I in chicken samples indicates high host-multiplicity and -adaptation of these genotypes.

Reports of *E. bieneusi* in horses are not too much. In current study, 11.5% of horses harbored *E. bieneusi,* which is close to the previous reports^61–64^, but lower than most of reports from China^65–67^. In Turkey, a country neighboring Iran, 18.7% of horses were detected positive for *E. bieneusi*^68^. *E. bieneusi* genotype horse1 thought to be a horse-specific genotype. This genotype was reported from horses in studies from Colombia^61^, Czech Republic^62,64^ Algeria^63^, and China^65,66^. However, this genotype is categorized in group 1, which is known as zoonotic group and might be an emerging zoonotic genotype in Iran. In addition, the genotype BEB6 was previously reported from horses in Turkey^68^ and China^65,67^. The presence of the genotype BEB6 in horses, chickens, cattle, and sheep in our study implies the non-host specificity of this genotype and high capability of the genotype BEB6 for adaptation in different hosts (Table 6).

**Table 6:** A summary of distribution of the genotypes D, BEB6, I, and horse1 from selected studied hosts (sheep, cattle, chicken, and horse) in the world.

No	Regions		Hosts	Number of samples/Pos	Genotypes (no.)	References
1	**Asia**	China	Sheep	260/237	BEB6 (237)	Ye et al. (2015)^69^
		China	Dairy cattle	1040/202	BEB6 (3), I (87)	Hu et al. (2017)^70^
		China	Sheep	318/20	BEB6 (12)	Qi et al. (2019)^71^
		China	Sheep	832/28	BEB6 (18)	Li et al. (2019)^72^
		China	Cattle (yak)	577/29	BEB6 (2), D (10), I (12)	Wu et al. (2019)^73^
		China	Sheep	414/177	BEB6 (60)	Shi et al. (2016)^74^
		China	Dairy cattle	879/214	I (61), BEB6 (17), D (2)	Li et al. (2016)^34^
		China	Horse	333/75	Horse 1 (37), D (3)	Deng et al. (2016)^66^
		China	Sheep	953/194	BEB6 (129)	Yang et al. (2018)^49^
		China	Dairy calves	388/61	D (3), Mixed of J and D (1)	Feng et al. (2019)^75^
		China	Sheep	620/93	BEB6 (6), I (1)	Chang et al. (2019)^76^
		China	Dairy cattle	3527/501	I (226), D (4)	Wang et al. (2019)^25^
		China	Chicken	206/4	D (2)	Cao et al. (2020)^56^
		China	Horse	262/81	BEB6 (9), D (1), horse1 (4)	Qi et al. (2016)^65^
		China	Cattle	314/31	I (1)	Zheng et al. (2020)^77^
		China	Sheep	1014/124	BEB6 (111)	Peng et al. (2019)^78^
		China	Dairy calves	514/85	I ( 19), D (2)	Qi et al. (2016)^79^
		China	Sheep	177/61	BEB6 (22)	Wu et al. (2018)^80^
		Northeast China	Dairy Cattle	133/40	I (2), D (1)	Zhao et al. (2015)^81^
		Northern China	Horse	325/24	Horse1 (8), BEB6 (2)	Li et al. (2020)^67^
		Northeast China	Cattle and sheep	1026/100	BEB6 (28), mixed of I and J (3) mixed of BEB6 and CM7 (5), mixed of BEB6 and NESH4 (3), mixed of BEB6 and NESH6 (1), mixed of BEB6 and OEB1 (5)	Jiang et al. (2015)^15^
		Southwestern China	Sheep	325/40	BEB6 (24)	Chen et al. (2018)^82^
		China	Sheep and Cattle (yak)	866/113	BEB6 (38)	Zhang et al. (2018)^48^
		China	Sheep	360/148	BEB6 (91)*	Zhang et al. (2020)^51^
		China	Sheep	138/31	BEB6 (15), D (6)	Zhao et al. (2015)^83^
		China	Cattle	93/35	I (8), mixed of I and J (5), mixed of I, J, and CHN1 (1)	Zhang et al. (2011)^44^
		China	Horse, Yak, Cattle, and Sheep	306/51	BEB6 (25)	Zhang et al. (2019)^39^
		Iran	Cattle	256/48	D (22)	Kord-Sarkachi et al. (2017)^84^
		Thailand	Cattle	60/3	D (3)	Udonsom et al. (2019)^27^
		South Korea	Cattle	538/80	I (3), D (1)	Lee et al. (2007)^85^
		South Korea	Cattle	180/15	I (7), D (1)	Lee et al. (2008)^86^
2	**Europe**	Czech Republic	Horse	66/34	D (34)	Wagnerová et al. (2012)^64^
		Czech Republic	Cattle	240/37	I (6)	Jurankova et al. (2012)^87^
		Sweden	Sheep (lambs)	72/49	BEB6 (32), mixed of BEB6 and OEB1 (4), mixed of BEB6 and OEB2 (4)	Stensvold et al. (2014)^47^
		Turkey	Horse	300/56	BEB6 (8)	Yildirim et al. (2020)^68^
		Germany	Cattle	28/3	I (2)	Rinder et al. (2000)^37^
		Germany	Cattle	60/7	I (1)	Dengjel et al. (2001)^88^
		Slovakia	Cattle	100/2	I (2)	Valenčáková et al. (2019)^89^
3	**Africa**	Algeria	Horse	219/15	horse1 (6), D (1)	Laatamna et al. (2015)^63^
		Algeria	Calves	102/11	BEB6 (2), I (1)	Baroudi et al. (2017)^90^
		Central Ethiopia	Sheep (lambs)	389/39	BEB6 (13)	Wegayehu et al. (2020)^50^
		South Africa	Cattle	50/9	I (1), D (1)	Abu Samra et al. (2012)^91^
4	**America**	Brazil	Dairy cattle	452/79	I (33), D (4), mixed I and BEB13 (1), mixed BEB4 and I (1)	da Silva Fiuza et al. (2015)^29^
		Brazil	Chicken	151/24	D (14)	da Cunha et al. (2015)^54^
		Brazil	Sheep	125/24	BEB6 (11), I (2)	da Silva Fiuza et al. (2016)^92^
		Argentina	Dairy cattle	70/10	I (2), D (1)	Del Coco et al. (2013)^36^
		USA	Dairy cattle	571/131	D (2)	Santín et al. (2005)^93^
		USA	Dairy cattle	990/239	I (134)	Santín et al. (2009)^94^
		USA	Horse	84/7	Horse1 (7)	Wagnerova et al. (2015)^62^
		USA	Horse	195/21	Horse1 (13), D (4)	Santín et al. (2010)^61^
		USA	Dairy cattle	47/17	I (17)	Fayer et al. (2012)^35^
		USA	Dairy cattle	819/285	I (59), mixed of I and BEB4 (3)	Santın et al. (2011)^32^
5	**Oceania**	Australia	Cattle	471/49	I (18)	Zhang et al. (2018)^30^

*Due to lack of access to supplementary tables, the number of the genotype BEB6 is attributed to all *E. bieneusi-*positive samples.

In studies that worked on several hosts, the number of samples, positive samples, and the genotypes were justified based on the only selected hosts (cattle, sheep, chicken, and horse) and investigated genotypes.

*Blastocystis* sp., was the most prevalent protozoan among samples 27.7% (48/173). *Blastocystis* sp. is a protist, which is frequently reported from humans^95,96^ and animals^97^. The prevalence rate of *Blastocystis* sp. in this study was higher than recent reports from Iran that indicated a rate of 14.98%^98^ among cattle, sheep, and, poultry, and 17.5%^99^ among dog and cat samples. Increasing evidence suggest the importance of animal to human transmission besides human to human transmission of *Blastocystis* sp. Until now, 17 genetic lineages (subtypes) have been confirmed together with recently five suggested subtypes^100^. In this study, ST1, ST5, ST7, ST10, and ST14 were reported from samples. Molecular epidemiology studies represented no host-specificity among subtypes, although some subtypes are frequently reported from certain hosts. In this study, ST1 and ST7 were the only human-prevalent subtypes, which were detected from cattle and chicken, respectively. ST1 was allele 4, which is commonly reported among ST1 isolated from humans, as well. This finding may highlight the importance of humans to animals and vice versa besides human to human transmission for this subtype. ST7 is an originally avian subtype, which has been reported from humans, as well^101–103^. Our finding showed that one of *Blastocystis* sp., isolated from chickens was ST7 allele 99. To our best of knowledge, allele 99 was only detected in a recent study by Mohammadpour et al. (2020)^99^ who characterized ST7 allele 99 from stool samples of dogs in south of Iran. The presence of avian subtypes such as ST7 among humans suggests the probability of zoonotic transmission from avian source^102,103^.

As result, ST10 was the major subtype identified in sheep and was also detected from cattle and a chicken. ST10 has been frequently reported from sheep and cattle with majority reports from sheep^98,104–106^. Notable, the presence of ST10 in chickens is not a common phenomenon and there is limited data on the report of this subtype in birds^98^. Although pseudoparasitism should be ruled out, cross-transmission of subtypes of *Blastocystis* sp. between different hosts appears to be a probable event. All ST10 in our study represents allele 152. Data on the allele distribution of ST10 is insufficient. In a most recent study, Mohammadpour et al. (2020)^99^, characterized allele 152 among stool samples from cats and dogs, which support the hypothesis suggesting cross-transmission of ST10 among animals. ST14 is a major subtype reported from sheep and cattle; however, there is no sufficient data on allele distribution of this subtype (Table 7).

**Table 7:** A summary of distribution of the subtypes 1, 5, 7, 10, and 14 from selected studied hosts (sheep, cattle, chicken, and horse) in the world.

No	Regions		Hosts	Number of samples/Pos	Subtypes (no.)	References
1	**Asia**	Iran	Chicken, sheep, cattle	395/115	1 (2), 5 (2), 7 (7), 10 (1), 14 (14)	Salehi et al. (2021)^107^
		Iran	Cattle, sheep, chicken	322/64	7 (15), 10 (31), 14 (15)	Rostami et al. (2020)^98^
		Iran	Cattle	75/11	5 (9), 10 (2)	Sharifi et al. (2020)^108^
		Malaysia	Cattle	80/35	1 (2), 5 (7), 10 (17), 14 (1)	Kamaruddin et al. (2020)^109^
		Malaysia	Cattle	3/1	10 (1)	Mohammad et al. (2018)^110^
		Malaysia	Chicken	104/27	1 (3), 7 (12)	Noradilah et al. (2017)^111^
		Malaysia	Chicken	179/47	1 (1), ST7 (5)	Farah Haziqah et al. (2018)^112^
		Lebanon	Dairy cattle	254/161	1 (9), 5 (3), 7 (1), 10 (55), 14 (46)	Greige et al. (2019)^104^
		Indonesia	Cattle	108/108	10 (20)	Suwanti et al. (2020)^113^
		Indonesia	Chicken	38/13	7 (8)	Yoshikawa et al. (2016)^114^
		Thailand	Cattle	42/21	10 (2)	Udonsom et al. (2018)^11^
		China	Cattle	526/54	5 (1), 10 (41), 14 (10)	Zhu et al. (2017)^115^
		China	Sheep	832/50	5 (8), 10 (25),14 (10)	Li et al. (2018)^105^
		China	Yak (cattle)	1027/278	10 (170), 14 (70)	Ren et al. (2019)^116^
		China	Cattle, sheep	256/20	1 (1), 5 (1), 10 (13), 14 (3)	Wang et al. (2017)^117^
		China	Chicken	46/6	7 (3)	Wang et al. (2018)^118^
		United Arab Emirates	Cattle, sheep	114/23	10 (7), 14 (3)	AbuOdeh et al. (2019)^106^
		South Korea	Cattle	1512/ 101	1 (6), 5 (5), 10 (9), 14 (10)	Lee et al. (2018)^119^
		Japan	Dairy cattle	133/57	10 (1), 14 (44)	Masuda et al. (2018)^120^
2	**Europe**	Turkey	Cattle	80/9	10 (2), 14 (7)	Aynur et al. (2019)^121^
		England	Cattle, sheep, chicken	46/29	1 (1), 5 (2), 7 (1), 10 (14), 14 (2), mixed type (9)	Alfellani et al. (2013)^122^
		Denmark	Cattle, sheep	NA	5 (3), 10 (23)	Stensvold et al. (2009)^123^
3	**Africa**	Libya	Cattle	36/15	ST5 (2), ST10 (6), ST14 (2), mixed type (5)	Alfellani et al. (2013)^122^
	**America**	USA	Cattle, chicken	36/8	10 (7), 7 (1)	Santín et al. (2011)^124^
		USA	Dairy cattle	47/9	10 (6), 14 (1), mixed type (2)	Fayer et al. (2012)^35^
		USA	Dairy calves	2539/73	ST5 (27), ST10 (5), ST14 (8)	Maloney et al. (2018)^125^
		Colombia	Cattle	25/20	1 (12)	Ramírez et al. (2014)^126^

NA: not assigned.

This table contains only those studies that amplified and sequenced the “barcoding region” of the SSU rRNA gene of *Blastocystis* sp.

In studies that worked on several hosts, the number of samples and the number of positive samples were justified based on the only selected hosts (cattle, sheep, chicken, and horse).

## Conclusion

The current study provides interesting data about the prevalence of *Blastocystis* sp., and *E. bieneusi* and their subtypes/genotypes among livestock. The presence of zoonotic potential genotypes of *E. bieneusi* in animals in this study increases the concerns on emerging microsporidia infections among humans who are in close-contact with livestock. Despite of levels of host-adaptation among the genotypes I, BEB6, and horse1 in our study, our findings propose high probability of cross-transmission of *E. bieneusi* among different hosts. In addition, characterization of zoonotic potential subtypes ST1 and ST7 among our samples provides a clue about the transmission dynamic of *Blastocystis* sp. between animals–animals and humans–animals, which needs further investigations with larger sample size.

## Materials and methods

### Ethics approval and consent to participate

Informed consent was taken from animal’s owners. Samples were taken during the veterinary medical care or checkup. All experimental protocols were approved by the Research Institute for Gastroenterology and Liver Diseases and all procedures performed in this study were approved by the ethical standards (IR.SBMU.RIGLD.REC.1398.033) released by Ethical Review Committee of the Research Institute for Gastroenterology and Liver Diseases, Shahid Beheshti University of Medical Sciences, Tehran, Iran. In addition, all methods were carried out in accordance with relevant guidelines and regulations, and all authors complied with the ARRIVE guidelines.

### Sample collection

**Figure 3:**
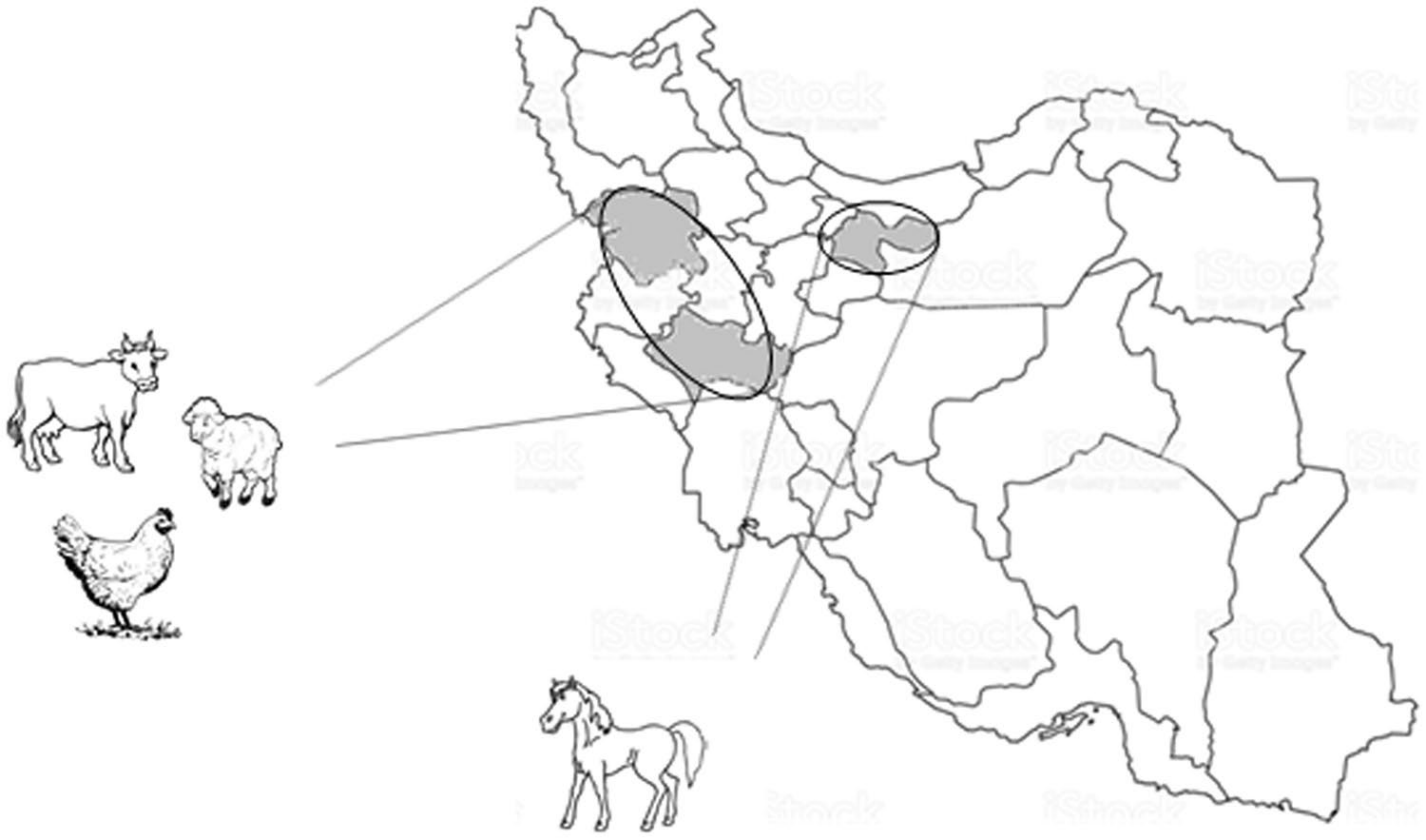
Iran map highlighting the sampling sites. The raw map was downloaded from free web source: https://commons.wikimedia.org/wiki/Atlas_of_the_world and edited with Photoshop cc by Hanieh Mohammad Rahimi.

A total of 173 stool samples were collected from domesticated animals including cattle, sheep, horse, and chickens. Stool samples of cattle, sheep, and chickens were from animal husbandry of two Kordestan and Lorestan provinces, western district of Iran. To avoid sampling biases, all samples of each farm were taken in one day. In addition, horse samples were collected from a horse riding club in Tehran province (Fig. 3). To avoid from sampling biases, all samples were collected from stalls before cleaning in one day. All samples were collected during Dec 2017 to Feb 2019. Samples were collected in appropriately sealed, labeled, and clean packages, and transported to the parasitology laboratory in the Research Institute for Gastroenterology and Liver Diseases, Shahid Beheshti University of Medical sciences, Tehran, Iran without preservative solutions. During 48 h after sampling, all samples were transferred to sterile 1.5 mL tubes and kept out in − 20 °C until DNA extraction and further analyses. DNA extraction was performed during the six months from sample collection.

### DNA extraction and purification

In order to extract total DNA from samples, aliquots of 250 mg (250 μL for liquid/diarrheic stools) of stool samples were placed in 1.5-mL tubes. In the case of formed samples, 250 mg of stool samples was suspended in one mL sterile PBS (pH = 7–8). Samples were centrifuged at 2500×*g* for 5 min, supernatant was discarded, and DNA was extracted from the remained pellet using stool DNA Extraction kit (Yekta Tajhiz Azma, Tehran, Iran). Finally, purified DNA was stored at − 20 °C until use in real-time PCR.

### Performing real-time PCR amplification

Four different specific primers targeting ribosomal genes of *Cryptosporidium* spp., *Blastocystis* sp., *E. bieneusi*, and *Encephalitozoon* spp., were selected (Table 8).

**Table 8:** Primers used in this study.

Target organism	Primer name	Primers sequence (5′ to 3′)	Approximate size of amplified fragment (bp)	Annealing (°C)	Target gene	References
*E. bieneusi*	EbITS-89F EbITS-191R	TGTGTAGGCGTGAGAGTGTATCTG CATCCAACCATCACGTACCAATC	103	60	Internal transcribed spacer (ITS)	^127^
*Encephalitozoon* spp.	MSP1F Eint227R	CACCAGGTTGATTCTGCCTGAC CTAGTTAGGCCATTACCCTAACTACCA	214*	60	Small subunit ribosomal RNA	^127^
*Cryptosporidium* spp.	JVAF JVAR	ATGACGGGTAACGGGGAAT CCAATTACAAAACCAAAAAGTCC	159	58	18S ribosomal RNA	^128^
*Blastocystis* sp.	BHRMF BHRMR	CGAATGGCTCATTATATCAGTT AAGCTGATAGGGCAGAAACT	220	60	18S ribosomal RNA	^129^

*The fragment size is different regarding the species.

Real-time PCR was carried out using Rotor-Gene Q (QIAGEN, Germany) real-time instrument. The real-time PCR reactions were conducted in a 15-μL total volume containing 7.5 μL of 2 × real-time PCR master mix (BIOFACT, Korea), 0.5 μL of each primer (5 ρM), 3.5 μL of distilled water, and 3 μL of template DNA. Amplification reactions were done as follows: 95 °C for 10 min followed by 40 cycles: 95 °C for 25 s, 59 °C for 30 s, 72 °C for 20 s, and ramping from 70 °C to 95 °C at 1°Cs^−1^. Appropriate positive sequenced controls for each parasite together with sterile distillated water as negative controls were tested in each run. The real-time PCR assays were carried out in duplicate to check the reproducibility. The melting profiles were also analyzed using Rotor-Gene Q software to exclude non-specific amplifications and primer-dimers.

Real-time PCR results were considered negative when the Ct value was more than 38 or no amplification curve was obtained. All samples with Ct value above 35 were either retested or their melting curve were justified by the positive control to confirm the result.

### Genotyping of *E. bieneusi*

To characterize the genotypes, Nested PCR was employed to amplify the ITS fragment of the rRNA gene of *E. bieneusi*, which were positive by the real-time PCR, as previously mentioned by Mirjalali et al. (2015)^43^. Primers EbGeno-Fe (5′-TTCAGATGGTCATAGGGATG-3) and EbGeno-Re (5-ATTAGAGCATTCCGTGAGG-3) were used to amplify a 465-bp fragment of the ITS gene. Afterward, EbGeno- Fi (5′-TCGGCTCTGAATATCTATGG-3′) and EbGeno-Ri (5′-ATTCTTTCGCGCTCGTC-3′) together amplified a 410-bp of the targeted fragment.

### *Blastocystis* subtyping

The barcoding region of *Blastocystis* sp. was amplified using primers RD5 (5′-ATCTGGTTGATCCTGCCAGT-3′) and BhRDr (5′-GAGCTTTTTAACTGCAACAACG-3′)^130^ in samples, which were positive using real-time PCR. Positive sequenced isolates of *E. bieneusi* and *Blastocystis* sp. together with sterile distillated water were included in each PCR run as positive and negative controls, respectively. To visualized the targeted fragments, 5 μL of PCR products was electrophoresed on 1.5% of agarose gel in TBE (0.09 M Tris, 0.09 M boric acid, 2 mM EDTA), stained with 0.5 μg/mL ethidium bromide, and visualized with UV transilluminator (Cleaver Scientific Ltd, Warwickshire, United Kingdom). All positive PCR products were sequenced using an ABI 3130 sequencer (Applied Biosystems, USA).

To characterize the genotypes and subtypes of *E. bieneusi* and *Blastocystis* sp., respectively, generated sequences were compared in the basic local alignment search tool (BLAST) search (http://www.ncbi.nlm.nih.gov/blast/) and then aligned and analyzed together with references orthologs, downloaded from the GenBank database, by the ClustalW program incorporated in BioEdit v. 7.2.6 software. Moreover, to obtain the alleles of *Blastocystis* sp. subtypes, the sequences of each subtype were subjected to typing tool (http://pubmlst.org/blastocystis/) database. The generated sequences were submitted in the GenBank database with accession numbers MW429392–MW429429 and MW426210–MW426254 for *E. bieneusi* and *Blastocystis* sp., respectively.

### Phylogenetic analysis

Phylogenetic trees were drawn for the ITS fragment and the barcoding region of *E. bieneusi* and *Blastocystis* sp., respectively, using the maximum-likelihood algorithm and Tamura-3-parameter substitution model in MEGAX software (http://www.megasoftware.net/)^131^. Bootstrap analyses with 1000 replications were employed to test the reliabilities of the trees. A number of reference sequences retrieved from the GenBank database were also included, alongside with our sequences to evaluate the phylogenetic relationships among isolates.

### Statistical analysis

Statistical analysis was performed using SPSS version 23 software (SPSS Inc., IBM, Chicago, IL, USA). Pearson’s chi-squared (χ^2^) for independence and Fisher’s exact two-sided tests were conducted to evaluate the prevalence and association between parasite and animals. A *P* value < 0.05 was considered statistically significant.

## Supplementary Information

Supplementary Legends.

Supplementary Figure 1.

Supplementary Figure 2.

Supplementary Figure 3.

## Data Availability

All generated data from the current study are included in the article and its supplementary materials and data.

## References

[1] Verweij JJ (2004). Simultaneous detection of *Entamoeba histolytica*, *Giardia lamblia*, and *Cryptosporidium parvum* in fecal samples by using multiplex real-time PCR. J. Clin. Microbiol..

[2] Li N (2012). Molecular surveillance of *Cryptosporidium* spp., *Giardia duodenalis*, and *Enterocytozoon bieneusi* by genotyping and subtyping parasites in wastewater. PLoS Negl. Trop. Dis..

[3] Andersen LO, Stensvold CR (2016). *Blastocystis* in health and disease: Are we moving from a clinical to a public health perspective?. J. Clin. Microbiol..

[4] Seguí R (2018). Prevalence of intestinal parasites, with emphasis on the molecular epidemiology of *Giardia duodenalis* and *Blastocystis* sp., in the Paranaguá Bay, Brazil: A community survey. Parasit. Vector.

[5] Javanmard E (2019). Molecular analysis of Blastocystis sp. and its subtypes from treated wastewater routinely used for irrigation of vegetable farmlands in Iran. J. Water Health.

[6] Muadica AS (2020). Molecular diversity of *Giardia duodenalis*, *Cryptosporidium* spp. and *Blastocystis* sp. in Asymptomatic School Children in Leganés, Madrid (Spain). Microorganisms.

[7] Karimi K (2020). Molecular epidemiology of *Enterocytozoon bieneusi *and *Encephalitozoon* sp., among immunocompromised and immunocompetent subjects in Iran. Microb. Pathog..

[8] Sow SO (2016). The burden of *Cryptosporidium* diarrheal disease among children < 24 months of age in moderate/high mortality regions of sub-saharan Africa and south Asia, utilizing data from the global enteric multicenter study (GEMS). PLoS Negl. Trop. Dis..

[9] Basak S, Rajurkar MN, Mallick SK (2014). Detection of *Blastocystis hominis*: A controversial human pathogen. Parasitol. Res..

[10] Yoshikawa H (2004). Fecal-oral transmission of the cyst form of *Blastocystis hominis* in rats. Parasitol. Res..

[11] Udonsom R (2018). *Blastocystis* infection and subtype distribution in humans, cattle, goats, and pigs in central and western Thailand. Infect. Gen. Evol..

[12] Peng XQ (2016). Infection rate of *Giardia duodenalis*, *Cryptosporidium* spp. and *Enterocytozoon bieneusi* in cashmere, dairy and meat goats in China. Infect. Gen. Evol..

[13] Delrobaei M (2019). Molecular detection and genotyping of intestinal microsporidia from stray dogs in Iran. Iran. J. Parasitol..

[14] Askari Z (2015). Molecular detection and identification of zoonotic microsporidia spore in fecal samples of some animals with close-contact to human. Iran. J. Parasitol..

[15] Jiang Y (2015). Zoonotic and potentially host-adapted *Enterocytozoon bieneusi* genotypes in sheep and cattle in northeast China and an increasing concern about the zoonotic importance of previously considered ruminant-adapted genotypes. Appl. Environ. Microbiol..

[16] Gill EE, Fast NM (2006). Assessing the microsporidia-fungi relationship: Combined phylogenetic analysis of eight genes. Gene.

[17] Fischer WM, Palmer JD (2005). Evidence from small-subunit ribosomal RNA sequences for a fungal origin of Microsporidia. Mol. Phys. Evol..

[18] Li W, Feng Y, Santin M (2019). Host specificity of *Enterocytozoon bieneusi* and public health implications. Trend Parasitol..

[19] Li W, Feng Y, Zhang L, Xiao L (2019). Potential impacts of host specificity on zoonotic or interspecies transmission of *Enterocytozoon bieneusi*. Infect. Gen. Evol..

[20] Claerebout E (2009). *Giardia* and other intestinal parasites in different dog populations in northern Belgium. Vet. Parasitol..

[21] Smith AF, Semeniuk CA, Kutz SJ, Massolo A (2014). Dog-walking behaviours affect gastrointestinal parasitism in park-attending dogs. Parasit. Vector.

[22] Osman M (2015). Prevalence and genetic diversity of the intestinal parasites *Blastocystis* sp. and *Cryptosporidium* spp. in household dogs in France and evaluation of zoonotic transmission risk. Vet. Parasitol..

[23] Yu Z (2018). Prevalence of intestinal parasites in companion dogs with diarrhoea in Beijing, China, and genetic characteristics of *Giardia* and *Cryptosporidium* species. Parasitol. Res..

[24] Incani RN (2017). Diagnosis of intestinal parasites in a rural community of Venezuela: Advantages and disadvantages of using microscopy or RT-PCR. Acta Trop..

[25] Wang HY (2019). Prevalence and population genetics analysis of *Enterocytozoon bieneusi* in dairy cattle in China. Front. Microbiol..

[26] Xue NY (2020). Molecular detection of *Cryptosporidium* spp. and *Enterocytozoon bieneusi* in Longjiang Wagyu cattle in northeastern China. Microb. Pathog..

[27] Udonsom R (2019). Identification of *Enterocytozoon bieneusi* in goats and cattle in Thailand. BMC Vet. Res..

[28] Bilgin T (2020). Molecular prevalence and phylogenetic characterization of *Enterocytozoon bieneusi* in healthy cattle. Turk. Parazitol. Derg..

[29] da Silva Fiuza VR, Lopes CW, de Oliveira FC, Fayer R, Santin M (2016). New findings of *Enterocytozoon bieneusi* in beef and dairy cattle in Brazil. Vet. Parasitol..

[30] Zhang Y, Koehler AV, Wang T, Haydon SR, Gasser RB (2019). *Enterocytozoon bieneusi* genotypes in cattle on farms located within a water catchment area. J. Eukaryot. Microbiol..

[31] Fayer R, Santín M, Trout JM (2007). *Enterocytozoon bieneusi* in mature dairy cattle on farms in the eastern United States. Parasitol. Res..

[32] Santín M, Dargatz D, Fayer R (2012). Prevalence and genotypes of *Enterocytozoon* bieneusi in weaned beef calves on cow–calf operations in the USA. Parasitol. Res..

[33] Tang C (2018). Genetic diversity within dominant *Enterocytozoon bieneusi* genotypes in pre-weaned calves. Parasit. Vector.

[34] Li J (2016). Occurrence, molecular characterization and predominant genotypes of *Enterocytozoon bieneusi* in dairy cattle in Henan and Ningxia, China. Parasit. Vector.

[35] Fayer R, Santin M, Macarisin D (2012). Detection of concurrent infection of dairy cattle with *Blastocystis*, *Cryptosporidium*, *Giardia*, and *Enterocytozoon* by molecular and microscopic methods. Parasitol. Res..

[36] Del Coco VF (2014). First report of *Enterocytozoon bieneusi* from dairy cattle in Argentina. Vet. Parasitol..

[37] Rinder H (2000). Close genotypic relationship between *Enterocytozoon bieneusi* from humans and pigs and first detection in cattle. J. Parasitol..

[38] Wang XT (2016). Multilocus genotyping of *Giardia duodenalis* and *Enterocytozoon bieneusi* in dairy and native beef (Qinchuan) calves in Shaanxi province, northwestern China. Parasitol. Res..

[39] Zhang Q (2019). *Cryptosporidium* spp., *Enterocytozoon bieneusi*, and *Giardia duodenalis* from animal sources in the Qinghai-Tibetan Plateau Area (QTPA) in China. Comp. Immunol. Microbiol. Infect. Dis..

[40] Yu F (2019). The potential role of synanthropic rodents and flies in the transmission of *Enterocytozoon bieneusi* on a dairy cattle farm in China. J. Eukaryot. Microbiol..

[41] Shen Y (2020). First identification and genotyping of *Enterocytozoon bieneusi* in humans in Myanmar. BMC Microbiol..

[42] Javanmard E (2020). The first report and molecular analysis of *Enterocytozoon bieneusi* from raccoon (*Procyon lotor*) in north of Iran. J. Eukaryot. Microbiol..

[43] Mirjalali H (2015). Genotyping and molecular analysis of *Enterocytozoon bieneusi* isolated from immunocompromised patients in Iran. Infect. Gen. Evol..

[44] Zhang X (2011). Identification and genotyping of *Enterocytozoon bieneusi* in China. J. Clin. Microbiol..

[45] Wang L (2013). Concurrent infections of *Giardia duodenalis*, *Enterocytozoon bieneusi*, and *Clostridium difficile* in children during a cryptosporidiosis outbreak in a pediatric hospital in China. PLoS Negl. Trop. Dis..

[46] Li W, Feng Y, Xiao L (2020). Diagnosis and molecular typing of *Enterocytozoon bieneusi*: The significant role of domestic animals in transmission of human microsporidiosis. Res. Vet. Sci..

[47] Stensvold CR, Beser J, Ljungström B, Troell K, Lebbad M (2014). Low host-specific *Enterocytozoon bieneusi* genotype BEB6 is common in Swedish lambs. Vet. Parasitol..

[48] Zhang Q (2018). *Enterocytozoon bieneusi* genotypes in Tibetan sheep and yaks. Parasitol. Res..

[49] Yang H (2018). Prevalence and genetic diversity of *Enterocytozoon bieneusi* in sheep in China. Parasit. Vector.

[50] Wegayehu T, Li J, Karim MR, Zhang L (2020). Molecular characterization and phylogenetic analysis of *Enterocytozoon bieneusi* in lambs in Oromia special zone, central Ethiopia. Front. Vet. Sci..

[51] Zhang Y (2020). *Enterocytozoon bieneusi* genotypes in farmed goats and sheep in Ningxia, China. Infect. Gen. Evol..

[52] Yildirim Y (2020). *Enterocytozoon bieneusi* in raw milk of cattle, sheep and water buffalo in Turkey: Genotype distributions and zoonotic concerns. Int. J. Food Microbiol..

[53] Reetz J (2002). First detection of the microsporidium *Enterocytozoon bieneusi* in non-mammalian hosts (chickens). Int. J. Parasitol..

[54] da Cunha MJ, Cury MC, Santín M (2016). Widespread presence of human-pathogenic *Enterocytozoon bieneusi* genotypes in chickens. Vet. Parasitol..

[55] Li W (2014). Genotypic distribution and phylogenetic characterization of *Enterocytozoon bieneusi* in diarrheic chickens and pigs in multiple cities, China: Potential zoonotic transmission. PLoS ONE.

[56] Cao S (2020). Prevalence and genetic characterization of *Cryptosporidium*, *Giardia* and *Enterocytozoon* in chickens from Ezhou, Hubei, China. Front. Vet. Sci..

[57] Ercan N, Duzlu O, Yildirim A (2020). Molecular detection and genotyping of microsporidia species in chickens in Turkey. Comp. Immunol. Microbiol. Infect. Dis..

[58] Javanmard E (2018). Molecular and phylogenetic evidences of dispersion of human-infecting microsporidia to vegetable farms via irrigation with treated wastewater: One-year follow up. Int. J. Hyg. Environ. Health.

[59] Tavalla M (2017). Molecular identification of *Enterocytozoon bieneusi* and *Encephalitozoon* spp. in immunodeficient patients in Ahvaz Southwest of Iran. Acta Trop..

[60] Tavalla M, Mardani-Kateki M, Abdizadeh R, Soltani S, Saki J (2018). Molecular diagnosis of potentially human pathogenic *Enterocytozoon bieneusi* and *Encephalitozoon* species in exotic birds in Southwestern Iran. J. Infect. Public Health.

[61] Santín M, Vecino JA, Fayer R (2010). A zoonotic genotype of *Enterocytozoon bieneusi* in horses. J. Parasitol..

[62] Wagnerová P (2016). *Cryptosporidium parvum* and *Enterocytozoon bieneusi* in American Mustangs and Chincoteague ponies. Exp. Parasitol..

[63] Laatamna AE (2015). Microsporidia and *Cryptosporidium* in horses and donkeys in Algeria: Detection of a novel *Cryptosporidium hominis* subtype family (Ik) in a horse. Vet. Parasitol..

[64] Wagnerová P (2012). *Enterocytozoon bieneusi* and *Encephalitozoon cuniculi* in horses kept under different management systems in the Czech Republic. Vet. Parasitol..

[65] Qi M (2016). *Enterocytozoon bieneusi* genotypes in grazing horses in China and their zoonotic transmission potential. J. Eukaryot. Microbiol..

[66] Deng L (2016). Molecular characterization and multilocus genotypes of *Enterocytozoon bieneusi* among horses in southwestern China. Parasit. Vector.

[67] Li F (2020). Zoonotic potential of *Enterocytozoon bieneusi* and *Giardia duodenalis* in horses and donkeys in northern China. Parasitol. Res..

[68] Yildirim A (2020). First report on the molecular prevalence of *Enterocytozoon bieneusi* in horses in Turkey: Genotype distributions and zoonotic potential. Parasitol. Res..

[69] Ye J (2015). Dominance of Giardia duodenalis assemblage A and *Enterocytozoon bieneusi* genotype BEB6 in sheep in Inner Mongolia, China. Vet. Parasitol..

[70] Hu S (2017). Zoonotic and host-adapted genotypes of *Cryptosporidium* spp., *Giardia duodenalis* and *Enterocytozoon bieneusi* in dairy cattle in Hebei and Tianjin, China. Vet. Parasitol..

[71] Qi M (2019). Distribution and molecular characterization of *Cryptosporidium* spp., *Giardia duodenalis*, and *Enterocytozoon bieneusi* amongst grazing adult sheep in Xinjiang, China. Parasitol. Int..

[72] Li WC, Wang K, Gu YF (2019). Detection and genotyping study of *Enterocytozoon bieneusi* in sheep and goats in east-central China. Acta Parasitol..

[73] Wu Y (2019). Molecular characterization and distribution of *Cryptosporidium* spp., *Giardia duodenalis*, and *Enterocytozoon bieneusi* from yaks in Tibet China. BMC Vet. Res..

[74] Shi K (2016). Molecular survey of *Enterocytozoon bieneusi* in sheep and goats in China. Parasit. Vector.

[75] Feng Y (2019). Prevalence and genotypic identification of *Cryptosporidium* spp., *Giardia duodenalis* and *Enterocytozoon bieneusi* in pre-weaned dairy calves in Guangdong, China. Parasit. Vector.

[76] Chang Y (2020). Molecular characterization of *Giardia duodenalis* and *Enterocytozoon bieneusi* isolated from Tibetan sheep and Tibetan goats under natural grazing conditions in Tibet. J. Eukaryot. Microbiol..

[77] Zheng XL (2020). Genotyping and zoonotic potential of *Enterocytozoon**bieneusi* in cattle farmed in Hainan province, the southernmost region of China. Parasite (Paris, France).

[78] Peng JJ (2019). Occurrence of *Enterocytozoon bieneusi* in Chinese Tan sheep in the Ningxia Hui autonomous region, China. Parasitol. Res..

[79] Qi M (2017). Dominance of *Enterocytozoon bieneusi* genotype J in dairy calves in Xinjiang, Northwest China. Parasitol. Int..

[80] Wu Y (2018). Occurrence and molecular characterization of Cryptosporidium spp., Giardia duodenalis, and Enterocytozoon bieneusi from Tibetan sheep in Gansu, China. Infect. Gen. Evol..

[81] Zhao W (2015). *Enterocytozoon bieneusi* in dairy cattle in the northeast of China: Genetic diversity of its gene and evaluation of zoonotic transmission potential. J. Eukaryot. Microbiol..

[82] Chen D (2018). Prevalence and multi-locus genotypes of *Enterocytozoon bieneusi* in black-boned sheep and goats in Yunnan Province, southwestern China. Infect. Gen. Evol..

[83] Zhao W (2015). Prevalence of *Enterocytozoon bieneusi* and genetic diversity of ITS genotypes in sheep and goats in China. Infect. Gen. Evol..

[84] Kord-Sarkachi E, Tavalla M, Beiromvand M (2018). Molecular diagnosis of microsporidia strains in slaughtered cows of southwest of Iran. J. Parasit. Dis..

[85] Lee JH (2007). Prevalence and molecular characteristics of *Enterocytozoon bieneusi* in cattle in Korea. Parasitol. Res..

[86] Lee JH (2008). Molecular detection of *Enterocytozoon bieneusi* and identification of a potentially human-pathogenic genotype in milk. Appl. Environ. Microbiol..

[87] Juránková J, Kamler M, Kovařčík K, Koudela B (2013). *Enterocytozoon bieneusi* in Bovine Viral Diarrhea Virus (BVDV) infected and noninfected cattle herds. Res. Vet. Sci..

[88] Dengjel B (2001). Zoonotic potential of *Enterocytozoon bieneusi*. J. Clin. Microbiol..

[89] Valenčáková A, Danišová O (2019). Molecular characterization of new genotypes *Enterocytozoon bieneusi* in Slovakia. Acta Trop..

[90] Baroudi D (2017). Molecular characterization of zoonotic pathogens *Cryptosporidium* spp., *Giardia duodenalis* and *Enterocytozoon bieneusi* in calves in Algeria. Vet. Parasitol. Reg. Stud. Rep..

[91] Abu Samra N, Thompson PN, Jori F, Zhang H, Xiao L (2012). *Enterocytozoon**bieneusi* at the wildlife/livestock interface of the Kruger National Park, South Africa. Vet. Parasitol..

[92] Fiuza V (2016). Zoonotic *Enterocytozoon bieneusi* genotypes found in Brazilian sheep. Res. Vet. Sci..

[93] Santín M, Trout JM, Fayer R (2005). *Enterocytozoon bieneusi* genotypes in dairy cattle in the eastern United States. Parasitol. Res..

[94] Santín M, Fayer R (2009). A longitudinal study of *Enterocytozoon bieneusi* in dairy cattle. Parasitol. Res..

[95] Deng L (2019). Epidemiology of *Blastocystis* sp. infection in China: A systematic review. Parasite (Paris, France).

[96] Javanmard E (2018). Impacts of human development index and climate conditions on prevalence of *Blastocystis*: A systematic review and meta-analysis. Acta Trop..

[97] Deng L (2021). First identification and molecular subtyping of *Blastocystis* sp. in zoo animals in southwestern China. Parasit. Vector.

[98] Rostami M (2020). Genetic diversity analysis of *Blastocystis* subtypes and their distribution among the domestic animals and pigeons in northwest of Iran. Infect. Gen. Evol..

[99] Mohammadpour I (2020). First molecular subtyping and phylogeny of *Blastocystis* sp. isolated from domestic and synanthropic animals (dogs, cats and brown rats) in southern Iran. Parasit. Vector.

[100] Stensvold CR, Clark CG (2020). Pre-empting pandora's box: *Blastocystis* subtypes revisited. Trend Parasitol..

[101] Greige S (2018). Prevalence and subtype distribution of *Blastocystis* sp. isolates from poultry in Lebanon and evidence of zoonotic potential. Parasit. Vector.

[102] Ramírez JD (2016). Geographic distribution of human *Blastocystis* subtypes in South America. Infect. Gen. Evol..

[103] Rezaei Riabi T (2018). Genetic diversity analysis of *Blastocystis* subtypes from both symptomatic and asymptomatic subjects using a barcoding region from the 18S rRNA gene. Infect. Gen. Evol..

[104] Greige S (2019). First report on the prevalence and subtype distribution of *Blastocystis* sp. in dairy cattle in Lebanon and assessment of zoonotic transmission. Acta Trop..

[105] Li WC, Wang K, Gu Y (2018). Occurrence of *Blastocystis* sp. and *Pentatrichomonas hominis* in sheep and goats in China. Parasit. Vector.

[106] AbuOdeh R (2019). Molecular subtyping of *Blastocystis* from diverse animals in the United Arab Emirates. Protist.

[107] Salehi R, Rostami A, Mirjalali H, Stensvold CR, Haghighi A (2021). Genetic characterization of *Blastocystis* from poultry, livestock animals and humans in the southwest region of Iran-Zoonotic implications. Transbound. Emerg. Dis..

[108] Sharifi Y (2020). Comparative genotyping of *Blastocystis* infecting cattle and human in the south of Iran. Comp. Immunol. Microbiol. Infect. Dis..

[109] Kamaruddin SK, Mat Yusof A, Mohammad M (2020). Prevalence and subtype distribution of *Blastocystis* sp. in cattle from Pahang, Malaysia. Trop. Biomed..

[110] Mohammad NA, Al-Mekhlafi HM, Anuar TS (2018). Subtype distribution of *Blastocystis* isolated from humans and associated animals in an indigenous community with poor hygiene in Peninsular Malaysia. Trop. Biomed..

[111] Noradilah SA (2017). Molecular epidemiology of *Blastocystis* sp in animals reared by the aborigines during wet and dry seasons in rural communities, Pahang, Malaysia. Southeast Asian J. Trop. Med. Public Health.

[112] Farah Haziqah MT (2018). Impact of pH on the viability and morphology of *Blastocystis* isolates. Trop. Biomed..

[113] Suwanti LT, Susana Y, Hastutiek P, Suprihati E, Lastuti NDR (2020). *Blastocystis* spp. subtype 10 infected beef cattle in Kamal and Socah, Bangkalan, Madura, Indonesia. Vet. World.

[114] Yoshikawa H (2016). Molecular survey of *Blastocystis* sp. from humans and associated animals in an Indonesian community with poor hygiene. Parasitol. Int..

[115] Zhu W (2017). First report of *Blastocystis* infections in cattle in China. Vet. Parasitol..

[116] Ren M (2019). First genotyping of *Blastocystis* in yaks from Qinghai province, northwestern China. Parasit. Vector.

[117] Wang J (2018). Subtype distribution and genetic characterizations of *Blastocystis* in pigs, cattle, sheep and goats in northeastern China's Heilongjiang province. Infect. Gen. Evol..

[118] Wang J (2018). Distribution and genetic diversity of *Blastocystis* subtypes in various mammal and bird species in northeastern China. Parasit. Vector.

[119] Lee H (2018). Occurrence and genetic diversity of *Blastocystis* in Korean cattle. Vet. Parasitol..

[120] Masuda A, Sumiyoshi T, Ohtaki T, Matsumoto J (2018). Prevalence and molecular subtyping of *Blastocystis* from dairy cattle in Kanagawa, Japan. Parasitol. Int..

[121] Aynur ZE (2019). Molecular characterization of *Blastocystis* in cattle in Turkey. Parasitol. Res..

[122] Alfellani MA (2013). Genetic diversity of *Blastocystis* in livestock and zoo animals. Protist.

[123] Stensvold CR (2009). Subtype distribution of *Blastocystis* isolates from synanthropic and zoo animals and identification of a new subtype. Int. J. Parasitol..

[124] Santín M, Gómez-Muñoz MT, Solano-Aguilar G, Fayer R (2011). Development of a new PCR protocol to detect and subtype *Blastocystis* spp. from humans and animals. Parasitol. Res..

[125] Maloney JG, Lombard JE, Urie NJ, Shivley CB, Santin M (2019). Zoonotic and genetically diverse subtypes of *Blastocystis* in US pre-weaned dairy heifer calves. Parasitol. Res..

[126] Ramírez JD (2014). *Blastocystis* subtypes detected in humans and animals from Colombia. Infect. Gen. Evol..

[127] Verweij JJ, Ten Hove R, Brienen EA, van Lieshout L (2007). Multiplex detection of *Enterocytozoon bieneusi* and *Encephalitozoon* spp. in fecal samples using real-time PCR. Diag. Microbiol. Infect. Dis..

[128] Jothikumar N, da Silva AJ, Moura I, Qvarnstrom Y, Hill VR (2008). Detection and differentiation of *Cryptosporidium hominis* and *Cryptosporidium parvum* by dual TaqMan assays. J. Med. Microbiol..

[129] Mohammad Rahimi H (2019). Development and evaluation of high-resolution melting curve analysis for rapid detection and subtyping of *Blastocystis* and comparison the results with sequencing. Parasitol. Res..

[130] Scicluna SM, Tawari B, Clark CG (2006). DNA barcoding of *Blastocystis*. Protist.

[131] Kumar S, Stecher G, Li M, Knyaz C, Tamura K (2018). MEGA X: Molecular evolutionary genetics analysis across computing platforms. Mol. Biol. Evol..

