# Supplementary material for: Molecular epidemiology and genotype/subtype distribution of Blastocystis sp., Enterocytozoon bieneusi, and Encephalitozoon spp. in livestock: concern for emerging zoonotic infections
Source: Sci Rep. 2021 Sep 1;11:17467. doi: 10.1038/s41598-021-96960-x (PMC8410837; doi:10.1038/s41598-021-96960-x)
Supplement: Supplementary file 1 — Supplementary Legends. [file 41598_2021_96960_MOESM1_ESM.docx]

**Supplementary figures**

**Supplementary Fig 1.** A) Cycling curves and B) melting curves generated from Blastocystis sp., E. bieneusi, Cryptosporidium spp. and Encephalitozoon spp. parasites.

**Supplementary Fig 2.** Cycling curves produced from A) *Blastocysti*s sp. B) *E. bieneusi* and C) *Encephalitozoon* spp. parasites.

**Supplementary Fig 3.** Melting curves generated from A) *Blastocysti*s sp., B) *E. bieneusi*, and C) *Encephalitozoon* spp. parasites in cattle, sheep, chicken, and horses.
