# Supplementary figures and images for: Molecular epidemiology and genotype/subtype distribution of Blastocystis sp., Enterocytozoon bieneusi, and Encephalitozoon spp. in livestock: concern for emerging zoonotic infections
Source: Sci Rep. 2021 Sep 1;11:17467. doi: 10.1038/s41598-021-96960-x (PMC8410837; doi:10.1038/s41598-021-96960-x)

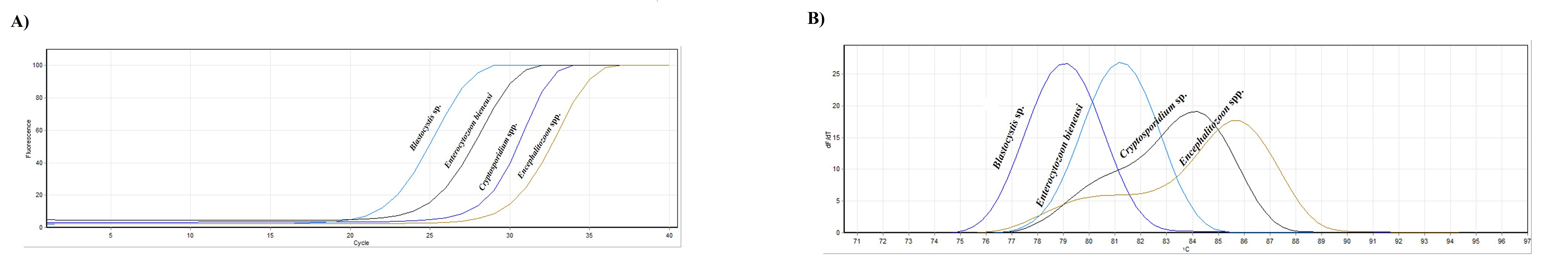

Supplement: Supplementary file 2 — Supplementary Figure 1. [file 41598_2021_96960_MOESM2_ESM.jpg]

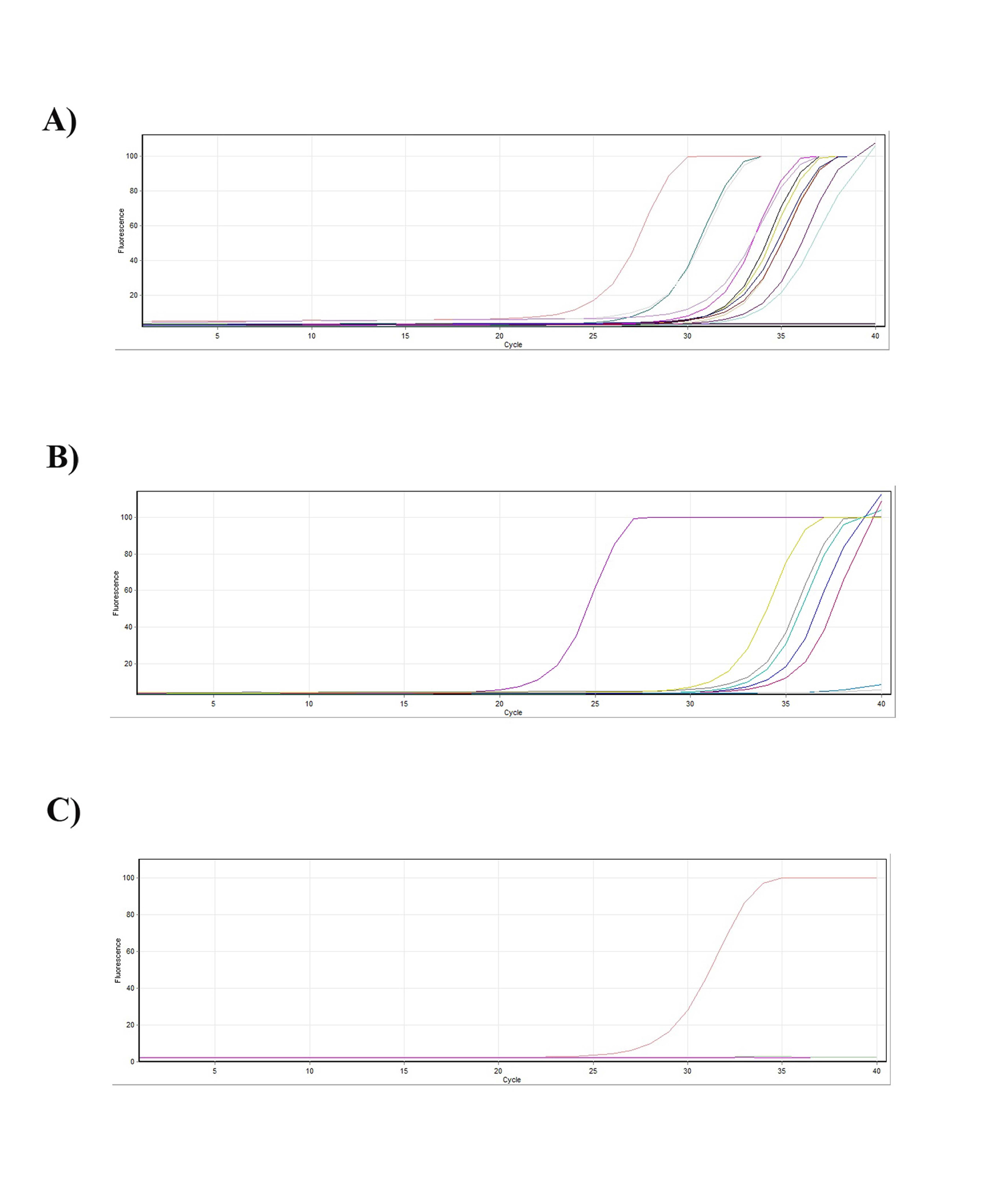

Supplement: Supplementary file 3 — Supplementary Figure 2. [file 41598_2021_96960_MOESM3_ESM.jpg]

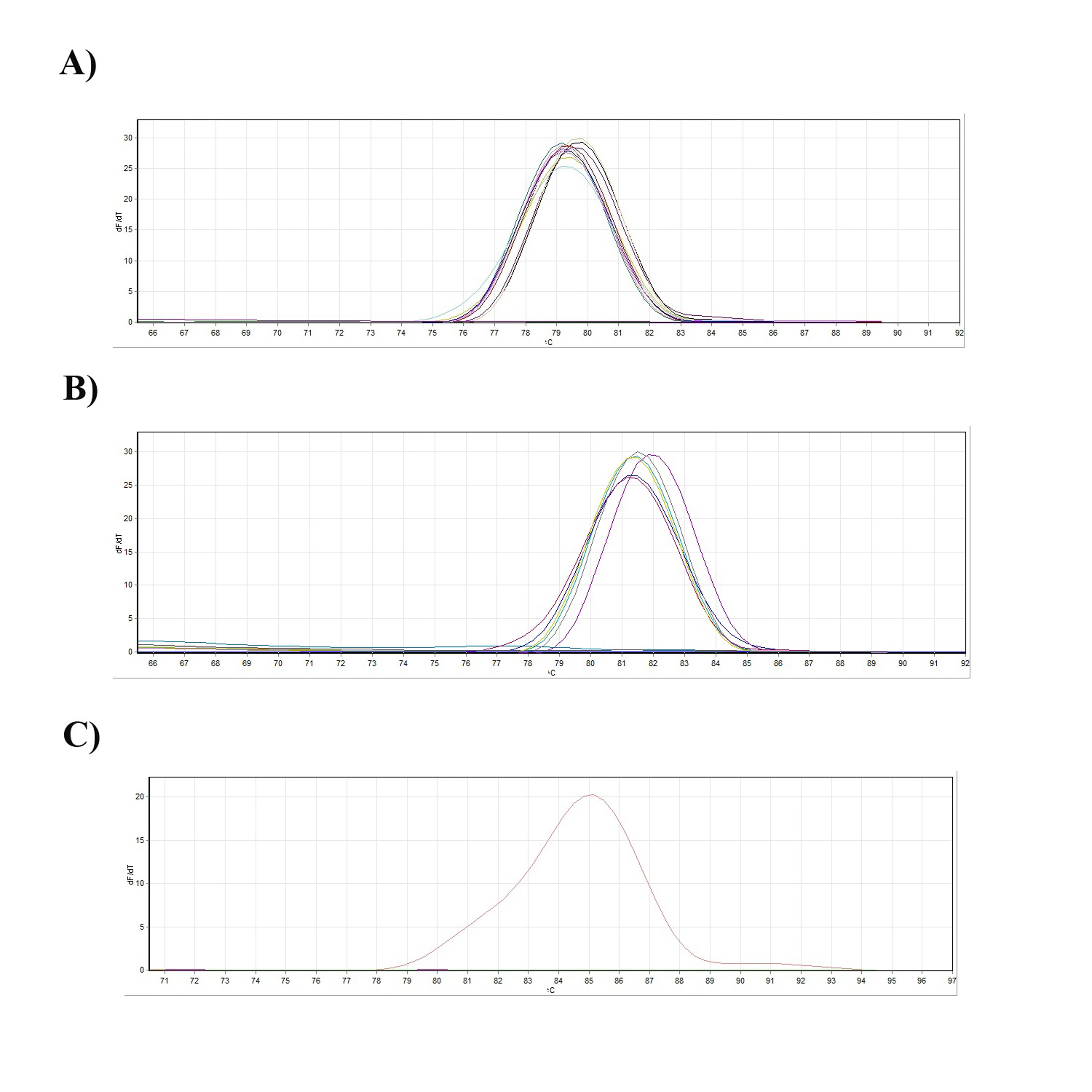

Supplement: Supplementary file 4 — Supplementary Figure 3. [file 41598_2021_96960_MOESM4_ESM.jpg]
